# Productive disruption: opportunities and challenges for innovation in infectious disease surveillance

**DOI:** 10.1136/bmjgh-2017-000538

**Published:** 2018-02-19

**Authors:** Caroline O. Buckee, Maria I E Cardenas, June Corpuz, Arpita Ghosh, Farhana Haque, Jahirul Karim, Ayesha S. Mahmud, Richard J Maude, Keitly Mensah, Nkengafac Villyen Motaze, Maria Nabaggala, Charlotte Jessica Eland Metcalf, Sedera Aurélien Mioramalala, Frank Mubiru, Corey M. Peak, Santanu Pramanik, Jean Marius Rakotondramanga, Eric Remera, Ipsita Sinha, Siv Sovannaroth, Andrew J Tatem, Win Zaw

**Affiliations:** 1 Department of Epidemiology, Center for Communicable Disease Dynamics, Harvard School of Public Health, Boston, Massachusetts, USA; 2 Centro de Atención y Diagnóstico de Enfermedades Infecciosas, Universidad Industrial de Santander, Bucaramanga, Colombia; 3 Epidemiology Bureau, Department of Health of the Philippines, Manila, Philippines; 4 Public Health Foundation of India, Vasant Kunj, New Delhi, India; 5 Programme for Emerging Infections (PEI), Infectious Diseases Division (IDD), ICDDR, B, Dhaka, Bangladesh; 6 Disease Control Department, Directorate General of Health Services, Dhaka, Bangladesh; 7 Mahidol-Oxford Tropical Medicine Research Unit, Faculty of Tropical Medicine, Mahidol University, Bangkok, Thailand; 8 Centre for Tropical Medicine and Global Health, Nuffield Department of Medicine, University of Oxford, Oxford, UK; 9 Dept of Ecology and Evolutionary Biology, Princeton University, Princeton, NJ, USA; 10 Division of Epidemiology and Biostatistics, Stellenbosch University, Stellenbosch, South Africa; 11 Infectious Disease Institute, College of Health Sciences, Makarere University, Uganda; 12 M&E Service, Data & Malaria Survey Division, National Malaria Control Program, Antananarivo, Madagascar; 13 Epidemiology Unit, Institut Pasteur, Madagascar; 14 Rwanda Biomedical Center, Kigali, Rwanda; 15 Technical Bureau, National Malaria Control Program, Pnom Penh, Cambodia; 16 WorldPop, Department of Geography and Environment, University of Southampton, Southampton, UK

**Keywords:** control strategies, epidemiology, health policy, health systems

**Summary box**

- New innovations that could transform infectious disease surveillance and control, including the use of Big Data, mobile health approaches and cutting edge quantitative methods, offer hope for disrupting traditional health systems and improving health worldwide.
- Much has been made of their potential, but very few have been translated successfully into policy or scaled up to a population level.
- We argue that there is currently a lack of integration of new approaches, making them unsustainable or unrealistic for most national control programmes and that the gulf between academia and policy makers remains a major barrier to their implementation.
- We propose that these innovations must be designed with direct input from national control programmes and embedded within already existing health systems.

## Background

Infectious diseases place an unacceptable and disproportionate social and economic burden on low-income countries. National disease control programmes have the difficult task of allocating limited budgets for interventions across regions of their countries, based on often disparate datasets of varying quality from a range of sources including clinics, hospitals, village health workers, the private sector and non-governmental organisations (NGOs). Every stage of the data collection and analysis pipeline for surveillance systems may be affected by a lack of capacity as well as by biases and misaligned incentives for reporting and managing data. Addressing these issues will be essential for effective reduction in the burden of endemic infectious diseases globally as well as to preparing for emerging epidemic threats.

Meanwhile, academic researchers—often in high-income settings—are developing increasingly sophisticated methods to collect and analyse data to improve spatial estimates of disease burden using new Big Data sources, mobile-Health or m-Health approaches or mechanistic and statistical modelling techniques. While these advances leap ahead, however, many remain most useful for estimating global disease distribution,^1^ rather than for national control programme prioritisation. Translating these new techniques to inform policy in endemic settings remains challenging. The pronounced disconnect between health systems and academia may limit the utility of new approaches. The high burden of work placed on healthcare workers in low-income settings further limits their scope and time available for engagement with methodological developments.

Despite ongoing challenges to implementation, however, there are promising analytical approaches that can leverage even patchy and low-quality data and diverse new data streams that can be productively harnessed to strengthen strategies for resource allocation when integrated with existing surveillance systems. We detail the data and analysis challenges faced by national disease control programmes, outline possible solutions offered by analytical approaches and new data-streams and conclude by outlining barriers to implementation.

## Challenges associated with infectious disease surveillance systems

**Figure 1:**
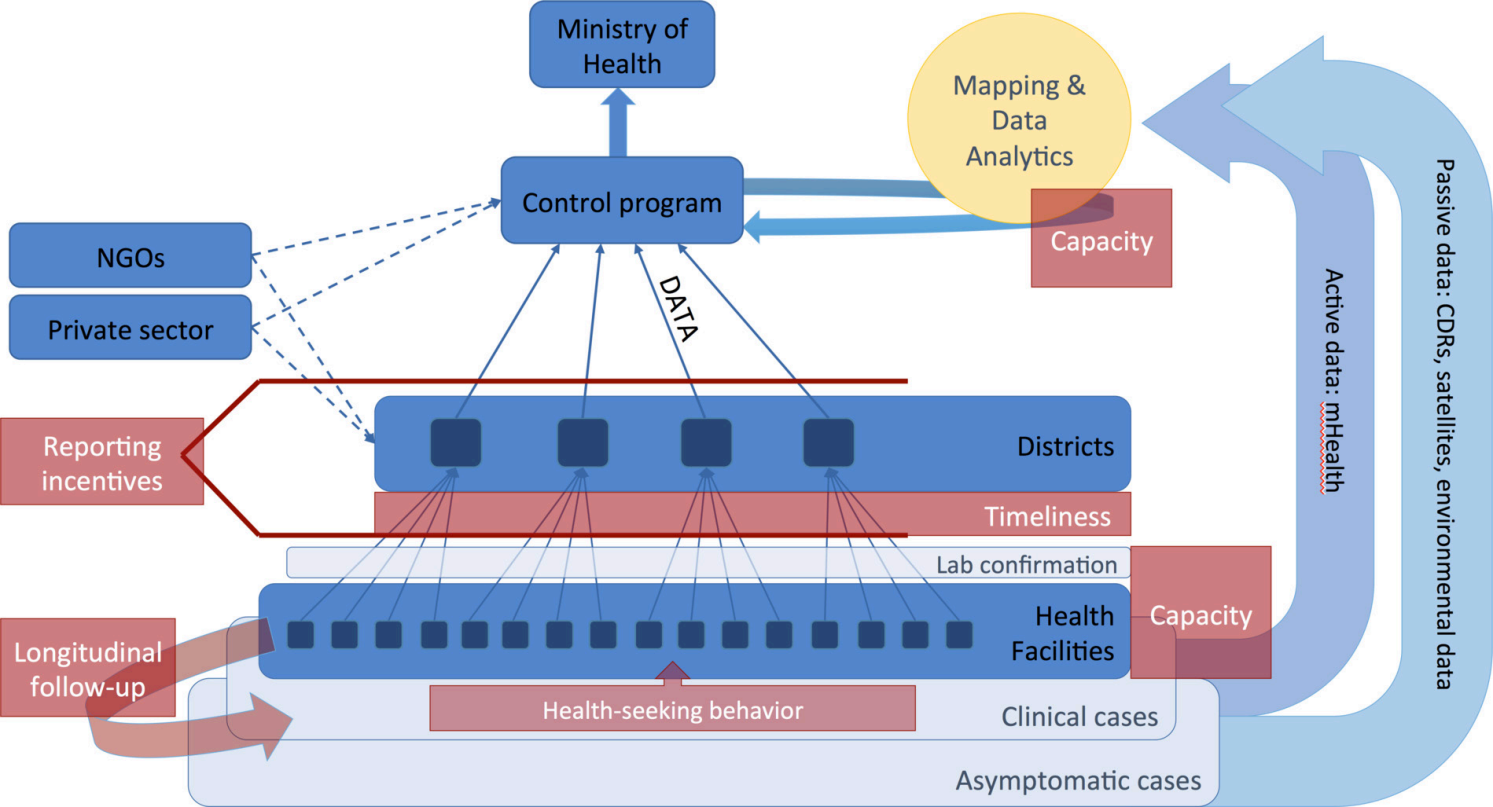
Data flows through health systems (blue) and major challenges faced by control programmes (red). A subset of clinical cases, which often represent only a subset of total infections both asymptomatic and clinical, are first detected by local health workers, most typically in health facilities and hospitals. Local health workers are also responsible for following up individuals with chronic infections requiring multiple treatments over months or years. Some fraction of clinical cases are lab confirmed, depending on capacity, and reported to regional or district centres, which in turn report to national control programmes. Data are often aggregated before being reported centrally. NGOs and the private sector may also produce a significant amount of epidemiological data. National control programmes aggregate and analyse data to map the distribution of disease burden, intervention efficacy and so on. New direct mHealth approaches (eg, participatory surveillance) and passively collected data (eg, from mobile phones via Call Data Records (CDRs); and satellites) may be used directly by control programmes to map underlying risks and population distributions. At every level, capacity remains an enormous issue for routine surveillance, and training for new approaches will be challenging for most control programmes. At different levels of the health system, incentives for reporting accurately may be misaligned, and timeliness of reporting may be particularly problematic for emerging threats. NGOs, non-governmental organisations.

Generally, epidemiological data about patients are reported by healthcare practitioners via passive surveillance systems to a central database, which is used to determine trends over time in and map the geographic distribution of burden of disease in different regions as well as the extent and efficacy of interventions (active surveillance via sentinel sites may also inform these efforts). These regional data in turn serve as an important basis for resource allocation decisions. Figure 1 illustrates the flow of data and potential hurdles faced by national control programmes and the ways in which new approaches may be used in parallel to traditional systems.

Data quality is often perceived as a major barrier to using passive surveillance data to guide resource prioritisation. Data may be delayed or simply missing from core settings and, even where available, is frequently under-reported, potentially in spatially and temporally variable ways.^2^ For example, a problematic reporting pattern that emerges repeatedly is an apparent increase in disease incidence that is actually caused by increased surveillance efforts and/or diagnostic capacity. Over-reporting is also a potential hazard when local regions are financially incentivised to exaggerate their needs. Further, since many local health centres lack diagnostic capabilities, much of the large-scale data rely on syndromic surveillance (influenza-like-illness, diarrhoea, fever) with low specificity. Additionally, estimates of the catchment population of health facility or district may be flawed, since data may be based on a single census from up to a decade ago, thus potentially both out of date^3^ and a poor reflection of seasonal fluctuations in population numbers.^2^ Collection of more detailed, highly curated data at sentinel sites seems promising, but may add little to national decision-making because such high-quality sites are likely to be limited in their spatial scale. All these issues mean that where case numbers exist, policy makers may have little confidence in them.

## Leveraging fragile data using statistical and mathematical principles

The first question is what can be done with existing infrastructure and data. With the right statistical or theoretical tools, even low-quality data can potentially be leveraged to help inform strategic deployment of control efforts. Thoughtful deployment of interpolation or geostatistical tools can be used to create smooth maps of burden or intervention efforts across space, also allowing extrapolation to unmeasured contexts.^1 4 5^ Autocorrelation models are also powerful tools, building on surveillance data to guide predictions about outbreaks of dengue, for example.^6^ Moving from statistical to mechanistic approaches, even if incidence reporting is erratic, dynamical signatures of the infectious process might still be detectable if additional data on features of cases are available, such as age, geographic location and gender. Age is a powerful covariate for infectious disease dynamics, as age of infection is linked to the magnitude of transmission. High-transmission pathogens often have a low average age of infection, as they move quickly through immunologically naïve populations.^7^ Conversely, implementation of control efforts is likely to increase the average age of infection,^8^ so intervention efficacy may be measured using shifting age structure of cases.

Where data are consistent through time, but not space, basic principles from infectious disease dynamics open the way to estimating characteristics of pathogens. For example, the growth rate of an epidemic can be extracted from incidence, allowing estimates of the net reproduction number, or R_0_, which captures the degree to which an outbreak is expected to grow (R_0_ >1) or shrink (R_0_ <1) (eg, deployed during the recent Ebola outbreak despite variation in reporting rates).^9^ Allocation of resources towards ‘source’ populations, where R_0_>1 then becomes possible—although maps of the locations and densities of rural populations (ie, denominator challenges) are also necessary. Infectious disease models can allow characteristics of the surveillance system, such as the magnitude of under-reporting, to be estimated where the susceptible population can be inferred (eg, via susceptible reconstruction).^10^ Where only syndromic surveillance is available, it may be possible to correct for background rates of focal syndromes to pull out the dynamics associated with a particular infection. This strategy has been used for influenza^11^ (influenza-like-illness data are frequently available but data on influenza are rare), enabling investigation of signatures of climate effects on the burden of infection,^12^ for example, which has potential to contribute to planning efforts. Alternatively, simulation tools based on known epidemiological parameters of particular pathogens can establish the degree to which the data are reliable and forecast outbreaks and emergence events and/or the impact of interventions like vaccination (eg, roll-out of cholera vaccination to contain an epidemic).^13^

## Complementing existing epidemiological information with new data sources

**Figure 2:**
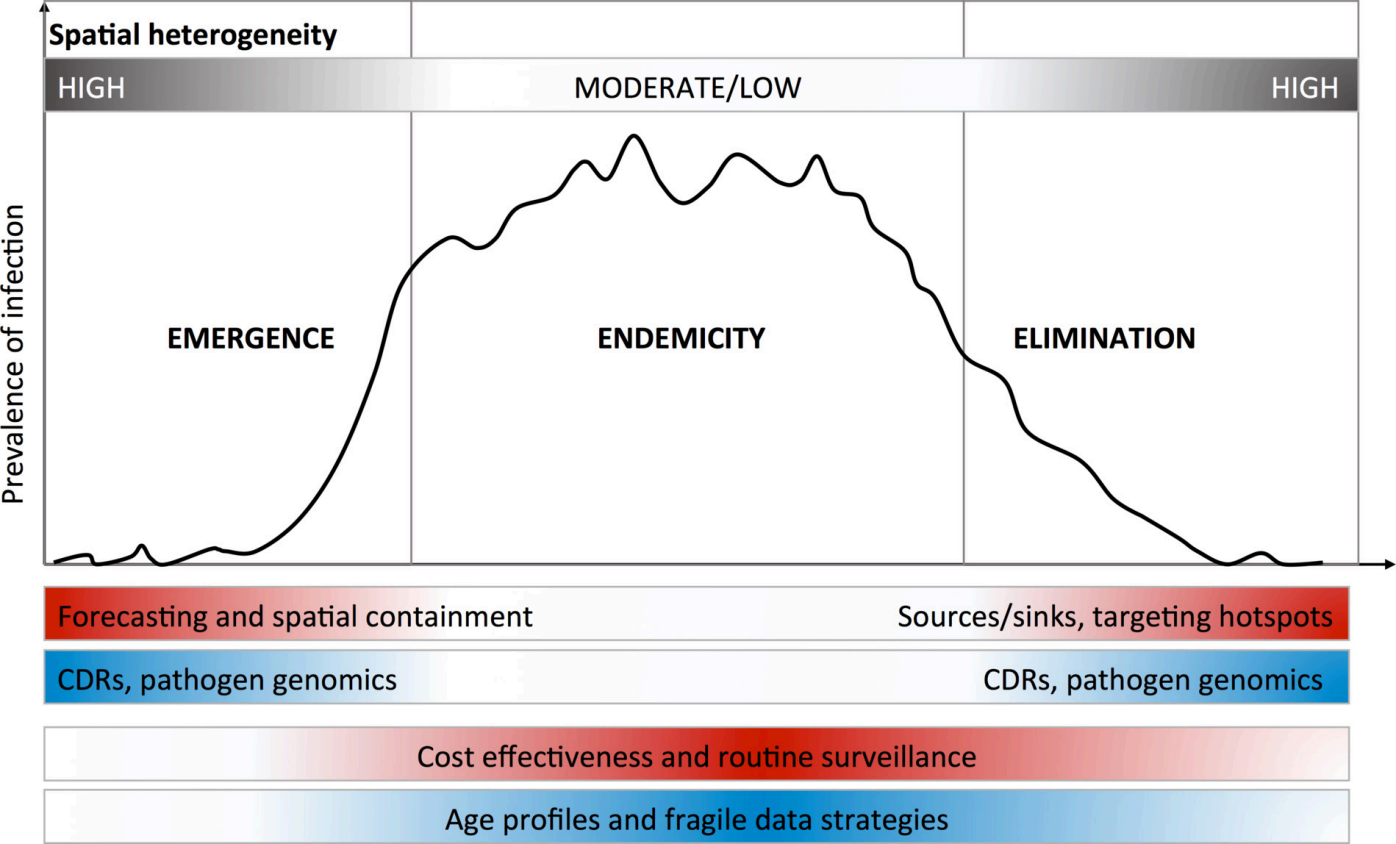
Optimal use of new approaches depends on epidemiological context. Different phases of epidemiological containment and control lend themselves to different analytical approaches and data sources. Here, we have highlighted the spatial dimensions of this issue, with emergence and elimination phases exhibiting high spatial heterogeneity. In these cases, pronounced heterogeneity produces signals in data that can be leveraged to model the spread of infection between populations. For endemic infections where prevalence is distributed throughout the country and controlling disease burden is the primary purpose of interventions, the use of age profiles of exposure and other analytical approaches may be used to enhance or make use of patchy or poor quality data.

While these analytical strategies can compensate for the limitations of different data quality issues, a range of promising new data streams are also available. Rapid technological advances make these increasingly affordable, offering additional or new data layers to include in epidemiological analyses.^1 14 15^ We focus on three new data types that are tractable in the context of control programme capacity: geospatial data, passively collected mobile phone records and pathogen genomic data. Note that we do not discuss the many mHealth approaches to actively engaging with populations directly, for surveillance or for interventions like health education,^16^ but these also provide data that do not rely on traditional surveillance systems. In general, different needs dominate at different phases across an epidemiological spectrum from emergence to elimination (figure 2), which will determine which data are useful and how they should be analysed.

*Geospatial data* are increasingly of high resolution and encompasses settlements and transport networks, indices of vegetation coverage, land use, land surface temperature and wind speed. These data can be combined with other geospatially referenced data such as meteorological data from weather stations, population densities or road networks to generate comprehensive estimates of environmental variables or indicators like remoteness or urbanicity relevant to communicable disease transmission. This can then be combined with point pattern data on vector presence via machine learning algorithms to determine features such as the likely range and local transmission intensity of vector borne infections like Zika virus.^17^

*Mobile phone data*—routinely collected by operators and providing information about the location and movements of subscribers in real time—offer tremendous promise for control programmes to measure disease spread, if appropriately anonymised and aggregated to protect subscriber privacy.^18^ Indeed, the development of appropriate anonymisation and aggregation protocols remains an important priority for academics and public health practitioners and will require carefully balancing the ethical risks of reidentifying individuals with the benefits of predicting disease spread and identifying targets for intervention. Integration of this information into risk mapping by control programmes offers particular promise where disease incidence is heterogeneous in time and space and mobility drives both changing burden of infection and the type of intervention needed. For example, during an emerging epidemic (eg, Ebola), spatial containment of the disease and proactive surveillance (in the correct locations) are essential, requiring specific spatial targets and estimates of how people will move the infection to new regions.^19^ At the other end of the spectrum, control programmes aiming for elimination (eg, malaria, measles) require accurate maps of remaining foci of transmission and an understanding of the relative importance of local versus imported cases of disease.^18^ These data can also be leveraged to address issues in estimation of the denominator or population at risk.^3^

*Genome sequencing* costs have declined strikingly in the last decades, making it increasingly feasible for control programmes to integrate pathogen sequencing and molecular epidemiology into their data collection and analysis strategies. Unlike mobile phone data, which offers an external view of mobility that can be used to model disease spread, analysis of pathogen genetic data provides complementary insights into transmission chains and pathogen gene flow between locations. During outbreaks, sequencing can provide insights into the place and time of the outbreak’s origin and the pace of its spatial spread.^20^ For elimination planning, pathogen sequencing can identify the regional or national origin of a particular isolate and estimate the rate of migration between populations where this information is difficult to measure by other means. For endemic pathogens like TB, where low incidence and relatively stable prevalence in many places makes analytical inferences and spatial data from mobile phones or satellites less tractable, genomics can provide key insights into the spread of drug resistance and the connectivity between different populations.

## Engaging community health workers and researchers who collect and analyse data is key

While our focus is on the potential of existing data despite perceived inadequacies and in use of novel data streams, it is important to note that country experience points to many low-cost adjustments to practice that could improve data availability. In multiple settings, there are important opportunities to share data across diseases, breaking down silos between disease surveillance and/or control programmes. This effort would be enhanced by electronic rather than paper-based reporting systems, allowing standardised data collection, aggregation and sharing. Perhaps most importantly, however, effectively engaging community health workers and others who collect and report patient data will be key to strengthening surveillance. For example, in Cambodia, malaria case reporting was improved when healthcare workers received feedback about the incidence of malaria in their area using colour-coded stickers; the effective use of mobile phones and tablets for reporting cases improved when health workers could use them for personal use and where internet coverage was restricted, the flexible reporting using mobile phones via SMS and later smartphones greatly improved timeliness of data collection.

It will be essential to sustain resources for engaging with health workers involved in generating and reporting surveillance data and not to impose technological advances at the expense of motivating and retaining qualified, reliable workers who form the foundation of an effective health system. Barriers to implementing approaches that use novel types of data or new analytical tools are rooted in human capacity (figure 1) at every level within control programmes, from local healthcare centres up to the ministry of health. While a considerable strength of novel data-streams such as satellite or mobile phone data are that they bypass many of the levels of reporting where data may be lost and incentives are misaligned, the insights they provide will be limited for setting public health agendas if they are not integrated with traditional reporting systems. For academics, one of the most important contributions in this regard will be providing training, so that public health officers have the technical capacity to understand the benefits of, and oversee the implementation of, new methods. Sustainable integrating of these approaches will require sustained commitment from Ministries of Health, donor agencies and academics.

## References

[1] Hay SI, George DB, Moyes CL, et al Big data opportunities for global infectious disease surveillance. PLoS Med 2013;10:e1001413 10.1371/journal.pmed.1001413 23565065 PMC3614504

[2] Buckee CO, Tatem AJ, Metcalf CJ Seasonal population movements and the surveillance and control of infectious diseases. Trends Parasitol 2017;33:10–20. 10.1016/j.pt.2016.10.006 27865741 PMC5423408

[3] Tatem AJ Mapping the denominator: spatial demography in the measurement of progress. Int Health 2014;6:153–5. 10.1093/inthealth/ihu057 25125576 PMC4161992

[4] Kraemer MU, Hay SI, Pigott DM, et al Progress and challenges in infectious disease cartography. Trends Parasitol 2016;32:19–29. 10.1016/j.pt.2015.09.006 26604163

[5] Takahashi S, Metcalf CJE, Ferrari MJ, et al The geography of measles vaccination in the African Great Lakes region. Nat Commun 2017;8:15585 10.1038/ncomms15585 28541287 PMC5458501

[6] Johansson MA, Reich NG, Hota A, et al Evaluating the performance of infectious disease forecasts: A comparison of climate-driven and seasonal dengue forecasts for Mexico. Sci Rep 2016;6:33707 10.1038/srep33707 27665707 PMC5036038

[7] Anderson RM, May RM Infectious diseases of humans. Oxford: Oxford University Press, 1991.

[8] Ferrari MJ, Grenfell BT, Strebel PM Think globally, act locally: the role of local demographics and vaccination coverage in the dynamic response of measles infection to control. Philos Trans R Soc Lond B Biol Sci 2013;368:20120141 10.1098/rstb.2012.0141 23798689 PMC3720039

[9] Shaman J, Yang W, Kandula S Inference and forecast of the current west african ebola outbreak in Guinea, sierra leone and liberia. PLoS Curr 2014;6 10.1371/currents.outbreaks.3408774290b1a0f2dd7cae877c8b8ff6 PMC4234409 25642378

[10] Bjørnstad ON, Finkenstadt B, Grenfell BT Endemic and epidemic dynamics of measles: estimating epidemiological scaling with a time series SIR model. Ecological Monographs 2002;72:169–84.

[11] Charu V, Zeger S, Gog J, et al Human mobility and the spatial transmission of influenza in the United States. PLoS Comput Biol 2017;13:e1005382 10.1371/journal.pcbi.1005382 28187123 PMC5349690

[12] Fisman DN, Tuite AR, Brown KA Impact of el niño southern oscillation on infectious disease hospitalization risk in the United States. Proc Natl Acad Sci U S A 2016;113:14589–94. 10.1073/pnas.1604980113 27791069 PMC5187703

[13] Azman AS, Luquero FJ, Rodrigues A, et al Urban cholera transmission hotspots and their implications for reactive vaccination: evidence from Bissau city, Guinea bissau. PLoS Negl Trop Dis 2012;6:e1901 10.1371/journal.pntd.0001901 23145204 PMC3493445

[14] Tatem AJ Mapping population and pathogen movements. Int Health 2014;6:5–11. 10.1093/inthealth/ihu006 24480992 PMC3989868

[15] Haque F, Ball RL, Khatun S, et al Evaluation of a smartphone decision-support tool for diarrheal disease management in a resource-limited setting. PLoS Negl Trop Dis 2017;11:e0005290 10.1371/journal.pntd.0005290 28103233 PMC5283765

[16] Tomlinson M, Rotheram-Borus MJ, Swartz L, et al Scaling up mHealth: where is the evidence? PLoS Med 2013;10:e1001382 10.1371/journal.pmed.1001382 23424286 PMC3570540

[17] Messina JP, Kraemer MU, Brady OJ, et al Mapping global environmental suitability for Zika virus. Elife 2016;5:e15272 10.7554/eLife.15272 27090089 PMC4889326

[18] Wesolowski A, Eagle N, Tatem AJ, et al Quantifying the impact of human mobility on malaria. Science 2012;338:267–70. 10.1126/science.1223467 23066082 PMC3675794

[19] Salje H, Lessler J, Endy TP, et al Revealing the microscale spatial signature of dengue transmission and immunity in an urban population. Proc Natl Acad Sci U S A 2012;109:9535–8. 10.1073/pnas.1120621109 22645364 PMC3386131

[20] Arias A, Watson SJ, Asogun D, et al Rapid outbreak sequencing of Ebola virus in Sierra Leone identifies transmission chains linked to sporadic cases. Virus Evol 2016;2:vew016 10.1093/ve/vew016 28694998 PMC5499387

